# Understanding the Potential of WO_3_ Based Sensors for Breath Analysis

**DOI:** 10.3390/s16111815

**Published:** 2016-10-29

**Authors:** Anna Staerz, Udo Weimar, Nicolae Barsan

**Affiliations:** 1 Institute of Physical and Theoretical Chemistry (IPTC), University of Tuebingen, Auf der Morgenstelle 15, D-72076 Tuebingen, Germany; anna.staerz@ipc.uni-tuebingen.de (A.S.); upw@ipc.uni-tuebingen.de (U.W.); 2 Center for Light-Matter Interaction, Sensors & Analytics (LISA+), University of Tuebingen, Auf der Morgenstelle 15, D-72076 Tuebingen, Germany

**Keywords:** WO_3_, breath analysis, gas sensors, SMOX based sensors

## Abstract

Tungsten trioxide is the second most commonly used semiconducting metal oxide in gas sensors. Semiconducting metal oxide (SMOX)-based sensors are small, robust, inexpensive and sensitive, making them highly attractive for handheld portable medical diagnostic detectors. WO_3_ is reported to show high sensor responses to several biomarkers found in breath, e.g., acetone, ammonia, carbon monoxide, hydrogen sulfide, toluene, and nitric oxide. Modern material science allows WO_3_ samples to be tailored to address certain sensing needs. Utilizing recent advances in breath sampling it will be possible in the future to test WO_3_-based sensors in application conditions and to compare the sensing results to those obtained using more expensive analytical methods.

## 1. Introduction

Breath analysis is an extremely interesting application field for gas sensors. The ability to monitor diseases and identify exposure to toxins via breath would revolutionize modern healthcare. It had been known since antiquity that human breath contains information about a person’s health, but it was not until the renaissance of analytical chemistry that the constituents of breath were really be identified [1]. In the 1970s Pauling et al. identified over 250 different substances using gas-liquid partition chromatography [2]. Today, with the help of modern technology, such as electrochemical and infrared detectors or sensitive mass spectrometers, thousands of different compounds have been identified in human breath [1]. As the analytical methods became more sensitive, the need for accurate sampling became apparent. In 1994, using an “alveolar gradient” (the difference between the amount in breath and in air), Phillips et al. tried for the first time to identify which compounds in human breath have an endogenous or exogenous origin [3,4]. As the origins of different biomarkers were discovered, it became clear that the breath sampling method must be customized for different biomarkers and should not influence the composition of the sample. Only once breath is properly sampled can a sensor quantitatively identify a specific biomarker. Today different commercially available breath samplers exist e.g., for NO or ethanol detection. These devices usually couple accurate samplers with sophisticated and expensive analytical methods. For widespread application, more compact and less expensive detector options are needed. Semiconducting metal oxide (SMOX)-based sensors are an attractive option for application in breath analysis devices because they are cheap, compact, robust, show high sensor responses, and allow for real-time measurements. Gaseous breath samples can be analyzed using SMOX-based sensors. Research has been conducted regarding the application of different oxides, e.g., ZnO for toluene [5] and SnO_2_/In_2_O_3_ [6] for ethanol. The review paper by Righettoni et al. provides a general overview of metal oxides´ potential use for breath analysis [7]. The high humidity (practically ~90%) and the presence of many interfering gases make breath analysis challenging. In 2012, despite the difficult conditions, Righettoni et al. reported the successful detection of acetone in human breath using a sensor based on Si-doped WO_3_ [8]. Today there is extensive research on WO_3_-based sensors for breath analysis. Through variation in doping, crystal structure, and morphology, changes in selectivity e.g., high sensor signals to different breath analysis relevant gases, are reported (see Table 1).

WO_3_ has fascinated scholars since the 18th century [37], and is widely used today [38]. In addition to its electrochromic and photocatalytic properties [38], WO_3_ is a commonly used material for commercial SMOX-based sensors. WO_3_ is an oxygen-deficient n-type semiconductor. Its resistance decreases when exposed to a reducing gas and increases in the presence of oxidizing gases [39]. Some basic research exists on the interaction of the WO_3_ surface with gases. For example, it has been shown using operando diffuse reflectance infrared (DRIFT) spectra conducted on sensors that CO reduces the WO_3_ lattice [40]:

(1) $$CO^{gas}+O_{O}\to CO_{2}^{gas}+V_{O}^{\cdot }+e^{-}$$

Akamatsu et al. examined and compared the surface reaction of WO_3_ with NO_2_ and NO using DRIFT spectroscopy on WO_3_ powder [41]. An oxidation of the surface was visible in the DRIFT spectra taken during exposure to NO_2_, indicating the following reaction [41,42]:

(2) $$NO_{2}^{Gas}+V_{O}^{\cdot }+e^{-}\to NO^{Gas}+O_{O}$$

A slight reduction was visible with NO [41]. Overall the sensor signals to NO_2_ were much higher than to NO. The competing surface reactions of NO with NO_2_ make sensing NO difficult. This example shows that it is important to consider not only how the test gases interact with the surface, but also to understand what effect surface reaction products may have on the sensor. In the case of H_2_S detection at low temperatures, the presence of atmospheric oxygen is necessary [43]. This is indicative of the oxygen-adsorbed model more closely described by Barsan et al. using the example of SnO_2_ [44]. Readers are referred to this article for more information on the general working principle of SMOX [44]. A more thorough examination and better understanding of surface reactions will enable a more deliberate and directed optimization of sensors in the future.

WO_3_ crystals are made up of corner-sharing WO_6_-octahedrals. The crystal structure of WO_3_ is temperature dependent, see Table 2 [38]. It has been reported that these phases are reversible [45,46,47]. In addition, a metastable hexagonal structure has been reported [48]. Different crystal structures can have different sensor characteristics, e.g., sensitivity and selectivity. In addition, like all SMOX-based sensors, the sensor characteristics of WO_3_ samples can be adjusted through different synthesis paths, resulting in morphology changes, etc. [39].

**Figure 1:**
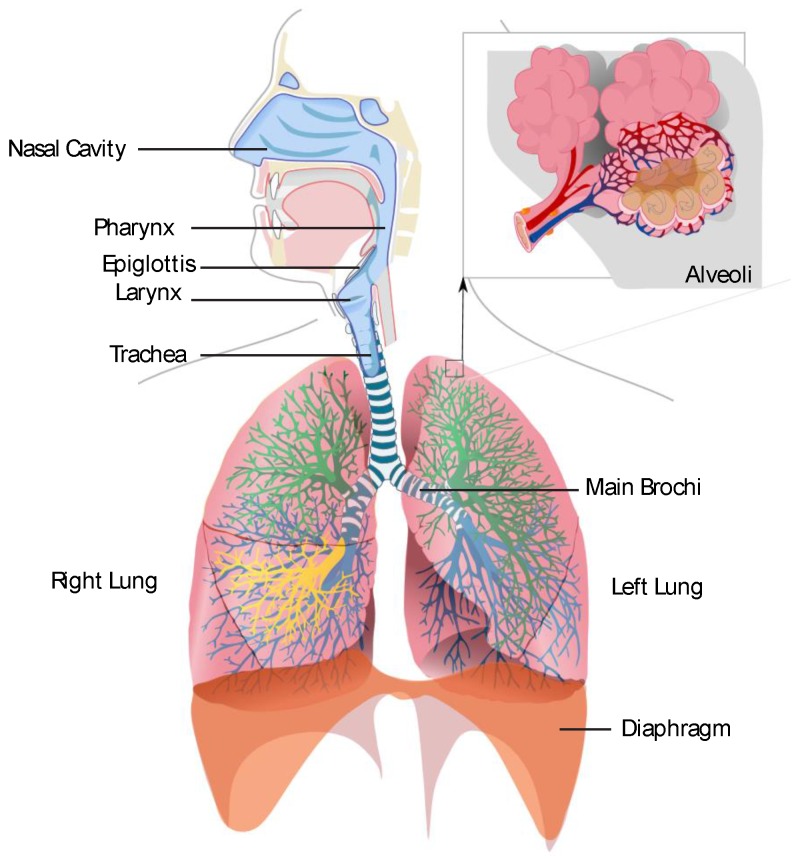
Basic picture of the respiratory system (Wikicommons: Respiratory).

A short review of the human respiratory system will illustrate the complexity of sampling human breath. During inhalation air enters through the mouth and nostrils into the pharynx, then passes the epiglottis into the trachea, and finally enters the bronchi which branch into bronchioles that end in clusters of alveoli (see Figure 1). The exchange of air with the bloodstream takes place in the alveoli [49]. The total exhaled breath contains a combination of the alveolar air and the air originating from the physiological dead space (air originating from the nasal/oral cavity, pharynx, larynx, trachea and bronchi). Depending on the origin of the biomarker, human blood or physiological dead space, different fractions of breath must be sampled. In the following different biomarkers will be examined. Existing sampling methods and possible coupling with WO_3_-based sensors will be described.

## 2. Biomarkers

### 2.1. Ethanol (C_2_H_6_O)

Today, one of the most commonly used breath analysis devices is the breathalyzer. It determines breath alcohol concentration (directly correlates to the blood alcohol level) [50]. Draeger is one of the largest suppliers of small portable breath alcohol detectors. Accurate determination of blood alcohol via breath analysis requires the alveolar air to be sampled [50]. Most of the Draeger systems have a plastic disposable mouthpiece into which the test person must blow evenly and without stopping for several seconds [50]. The mouthpiece measures the temperature and the volume of breath. A certain volume and flow rate, dependent on the age and sex of the test person, is necessary before a measurement can be completed [50]. More information about the Draeger samplers can be found on the Draeger homepage. The sampling method coupled with an electrochemical sensor gives accurate results [51].

Breathalyzers, for personal use, that couple alveolar breath samplers with SMOX-based sensors are commercially available [52]. In 2015, Rosenberg et al. compared the AL5500 from ACE Instruments (Freilassing, Germany), a detector containing a SMOX-based sensor, to 10 different fuel cell-based analyzers [52]. Overall, the AL5500 was more accurate than approximately half and more precise than two of the other analyzers (see Table 3) [52]. The SMOX-based detector is by far the cheapest of the commercially available alcohol testers. This difference in cost is substantial as small portable inexpensive personal sensors become more marketable (see Table 3).

Current research focuses on tailoring SMOX materials, in general, to better detect ethanol in breath [6,53,54]. Research exists on the suitability of WO_3_-based sensors for breath alcohol detection, see Table 4. Sensors based on pure WO_3_ respond to the presence of ethanol [9,10]. The selectivity, sensitivity, and stability of SMOX-based sensors are dependent on the material design [55]. Dopants can be used to alter the sensor characteristics of SMOX. Labidi et al. found that doping with Au increased the response of the WO_3_-based sensors to ethanol, while it decreased as a result of Pd doping [9]. Nayak et al. found that sensors based on WO_3_-(0.54)SnO_2_ nanostructures show better sensor signals than those made from pure WO_3_ and SnO_2_ [11]. In addition the sensors are also reportedly selective to ethanol in the presence of other volatile organic compounds, e.g., ammonia, acetone, chloroform, hexane, and toluene [11]. Another interesting method for increasing the sensor response of SMOX-based sensors is fluctuation-enhanced sensing (FES). Irradiation with ultraviolet (UV) light has a photocatalytic effect on resistive gas sensors [56]. Through irradiation of Au-NP decorated WO_3_ with two different lasers it is possible to increase the sensor signals to both low (>20 ppm) and higher (>50 ppm) ethanol concentrations [56]. The effect of humidity on the sensor response was not considered in any of the research. This aspect is highly important for medical diagnostics as human breath is saturated with humidity. It will have to be examined and addressed in the future. Using modern nanoscience, material synthesis can be better controlled at a molecular level [57]. As the field advances, it will be possible to lower power requirements, and to raise the sensitivity and selectivity of materials [57]. In the future, WO_3_-based sensors can be more ideally tailored for ethanol detection in breath. The commercially available breath alcohol detectors provide a proof of concept that SMOX-based sensors can successfully be coupled with existing breath samplers. Although the concentrations of most other biomarkers in breath are lower (see Table 1), experience with the sampling and sensing of ethanol provides a good foundation for the sensing of other gases in breath.

### 2.2. Acetone (C_3_H_6_O)

Patients with diabetes are either unable to make or effectively use insulin. As a result their body burns fat instead of glucose for energy leading to heightened acetone concentrations in blood [19]. As with ethanol, the concentration of acetone in the alveolar breath reflects the arterial concentration [58]. Deng et al. examined the concentration of acetone in human breath using gas-chromatography-mass-spectroscopy (GC-MS). Sick patients were found to have a concentration of acetone between 1.76 and 3.73 ppmv. In the healthy control group the range was much lower, between 0.22 and 0.80 ppmv [19].

**Figure 2:**
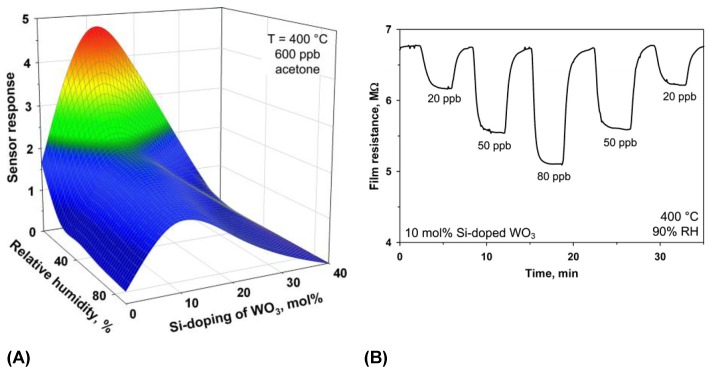
(**A**) The response of WO_3_-based sensors at 400 °C to 600 ppb of acetone are depicted with respect to Si-content and relative background humidity. A sensor based on 10% mol Si-doped WO3 is optimal. (**B**) Using this sensor it was possible to detect ultralow concentrations of acetone (20–90 ppb) in 90% RH [16]. The figure is reprinted from [16]. Copyright 2010 American Chemical Society.

In 2015 Righettoni et al. coupled a sensor based on an ε-phased Si-doped WO_3_ with a sophisticated breath sampler marketed under the name SOFIA) from the company Loccioni, Angeli di Rosora, Italy [13]. The sampler was similar to those used for ethanol detection. The end tidal and dead volume fractions of the breath are distinguished based on the CO_2_ concentration [13]. The sampler has two major components, the handheld sensing and sampling unit (SSU) and the portable process control unit (PCU). For more details and pictures of the sampler, readers are referred to the research of Righettoni et al. [13] or the homepage of Loccioni (http://humancare.loccioni.com). The repeated results from individual subjects were similar. The SOFIA breath sampler is modular and further metal oxide gas sensors could easily be integrated [13]. This is important for future research, because numerous other WO_3_ samples show promise as acetone sensors, see Table 5 For example, Li et al. reported that sensors based on WO_3_ hollow spheres have much better responses to organic gases than sensors made from traditional WO_3_ particles [14]. The sensors based on the hollow spheres from Li et al. were, however, not selective and also showed high signals to ethanol, CS_2_, and other organic gases [14]. In 2014, Choi et al reported that Rh loading increased the sensor response and selectivity of these WO_3_ hollow spheres to acetone. The effect of humidity on the sensor responses was also decreased through Rh loading [15]. Rhodium is believed to influence the surface reactions during sensing. Due to its strong affinity for water, rhodium is believed to suppress the reaction between the sensing surface and humidity, which explains why the sensor response is no longer humidity dependent [15]. In addition to changing the surface reactions, dopants are reported to alter basic material characteristics. Like Righettoni et al., Wang et al. reported the stabilization of ε-WO_3_ above room temperature using dopants [16,17]. The results of Righettoni et al. are shown in Figure 2. Wang et al. used Cr to stabilize the ε-phase, which is usually only stable below −40 °C, at RT. The addition of Cr is reported to introduce distortions into the WO_3_ matrix repelling the tungsten atoms from the centric positions in the octahedral WO_6_. Cr-doped (10 at%) WO_3_ showed high signals and selectivity to acetone, and against ethanol, methanol, NO, NO_2_, NH_3_, and CO. The acentric structure of the ε-phase is thought to be highly selective to acetone, [17].

Recently, methods have been developed to better control and optimize doping, for example by Kim et al. who created sensors based on Pt-functionalized WO_3_ [18]. It is known that through the agglomeration of the catalytic nanoparticles, the sensing properties decrease. Kim et al. encapsulated the Pt nanoparticles in an apoferritin template to prevent large agglomerates. Using this method they attained a very narrow size distribution for the nanoparticles, between 1 and 3 nm [18]. The sensors showed high signals to acetone and low signals to many other breath analysis relevant gases, e.g., H_2_S, toluene, ethanol, and ammonia. The high humidity of human breath was taken into consideration, and all measurements were done at 90% RH [18].

Righettoni et al. coupled the sophisticated breath sampler with Si-doped WO_3_ to detect acetone as a proof of concept [13]. In the future additional measurements can be done using this modular sampling device to examine which samples of WO_3_ are best suited to detect acetone in breath. Furthermore, the sampler can be used to examine the breath analysis of other biomarkers present in alveolar air, e.g., ammonia and toluene.

### 2.3. Ammonia (NH_3_)

Davies et al. found that heightened concentrations of ammonia in human breath are characteristic of end stage renal failure [25]. Urea accumulates in the blood as a result of kidney failure [59]. In the gastrointestinal tract the excess urea is then decomposed into ammonia [59]. Saliva bacteria can also decompose excess urea [59], presenting the possibility of contamination. The ammonia concentration in the alveolar air in patients suffering from renal disease is in the ppm range [25].

WO_3_ based samples show promise for use in breath ammonia detection, see Table 6. As early as 1992, Maekawa et al. reported that Au-doped WO_3_ samples show good signals to NH_3_ [20]. The measurements were done in air and the best results were attained with sensors operated at 450 °C. Sensors based on their Au-loaded sample showed better responses to NH_3_ than sensors based on pure WO_3_, as well as samples doped with Rh, Ru, Pd, and Ag. No characterization of the material was published [20], so no information is present on the crystal phase of the sample or how exactly the sample was doped.

In 2000, Llobet et al. reported for the first time that sensors based on pure WO_3_ respond to ammonia. When operated at 300 °C, the sensors were selective to ammonia in the presence of toluene, ethanol, and benzene. The base resistance of the samples was found to change as a result of background humidity; the effect on the sensor response was not evaluated. The crystal phase of the WO_3_ samples was not determined [21]. Recently, however, Takács et al. and Wang et al. reported that the metastable hexagonal phase of WO_3_ shows very good sensor responses to ammonia [22,23]. Wang et al. report that through acid precipitation, it is possible to synthesize the metastable hexagonal structure. Under the same sensing conditions the signals of sensors based on the hexagonal sample were higher than those based on γ-WO_3_ (monoclinic) [23]. Takács et al. synthesized h-WO_3_ samples with different morphologies [22]. The nanorods showed better responses than the carnation-formed particles [22]. The decrease in the resistance of WO_3_-based sensors is caused by the NH_3_ oxidation on the surface of the metal oxide grains [60]. A possible oxidation product of NH_3_ is NO [61]. NO can react to NO_2_ in the presence of atmospheric oxygen. WO_3_ is known to respond to the NO_2_ [41]. Jiménez et al. reported signal instability as a result of the ammonia oxidation and the NO_2_ adsorption, i.e., reduction [24]. Epifani et al. was able to eliminate the signal instability by measuring at higher temperatures and through loading with Cr [60]. Epifani et al. reports better sensor responses for sensors based on Cr-doped WO_3_ than those based on pure WO_3_. Based on X-ray diffraction (XRD) measurements it was determined that the WO_3_ sample is in the δ monoclinic phase and that the Cr is predominantly interstitially incorporated [60]. In addition to catalyzing the oxidation of NH_3_, interstitial Cr decreased the number of oxygen vacancies, lessening the adsorption sites of NO_2_ [60]. This is a perfect example of how advancements in synthesis methods have allowed researchers to address issues by ‘‘tuning’’ materials.

The reactivity of NH_3_ makes sampling particularly challenging [62]. A modular breath sampler from Loccioni, Angeli di Rosora, Italy was used. Solga et al. found that for reproducible results of exhaled breath ammonia, the temperature of the breath sampler, the mode of breathing, and mouth pH must be controlled [62]. For more details on the difficulty of detection NH_3_ in breath please refer to the work by Solga et al. [62]. Although the results were promising, there are important limitations. In particular, the study was done on only one subject and the role of diet or testing time was not considered [62].

In the case of exhaled NH_3_, an optimal sampling protocol must still be developed and tested. Only once sampling is accurate and reproducible will it be possible to examine if and which sensors based on WO_3_ are optimal for exhaled NH_3_ monitoring.

### 2.4. Toluene (C_7_H_8_)

**Figure 3:**
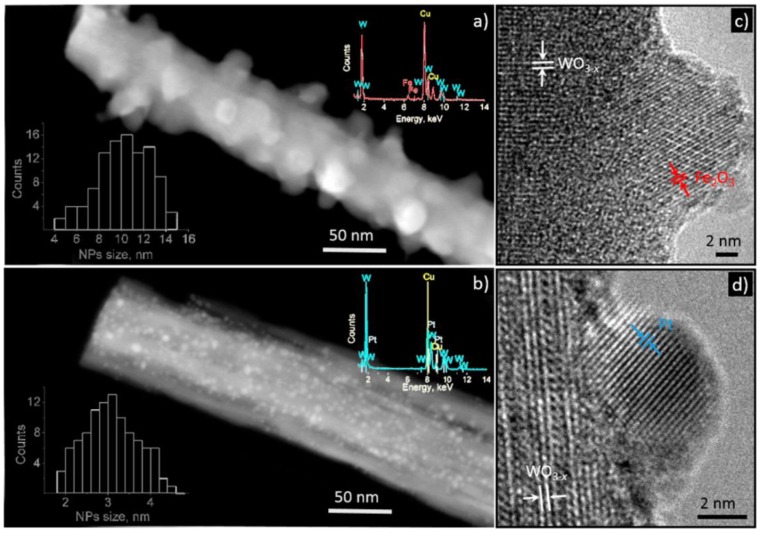
STEM and HRTEM of the WO_3−x_ nanoneedles functionalized with Fe_2_O_3_. The figure is reprinted with permission from [27]. Copyright 2015 American Chemical Society.

Biomarkers in breath can not only be used to monitor diseases but also to detect exposure to hazardous substances. Toluene is a commonly used colorless industrial solvent that has neurotoxic and narcotic properties [63]. The Occupational Safety and Health Administration of the United States (OSHA) has limited toluene exposure to an 8-h weighted average of 200 ppm (1910.1000 Table Z-2). Carlson et al. report that roughly 28% of the inspired toluene concentration is still present 30 min after exposure in the alveolar air [64]. This would be over 56 ppm if toluene concentrations over the OSHA limit are present. Sensors based on differently modified WO_3_ samples are reported to show good signals to toluene within the relevant concentration range [26,27], see Table 7. For example, Miao et al. report that the sensors based on anisotropic WO_3_·H_2_O nanoplatelets operated at 300 °C show high signals to toluene (between 10 and 100 ppm) [26]. The nanoplatelets were synthesized using a hydrothermal synthesis with malic acid. The anisotropic nanoplatelets which have more of the (0 1 0) facet, show higher responses to toluene than the less anisotropic nanocubes. The explanation provided by the authors theorizes that during the adsorption of toluene on the (0 1 0) facet of orthorhombic WO_3_·H_2_O more electrons are extracted from the surface than through adsorption on the (1 0 0) and (0 0 1) facets [26]. Different dopants have also been reported to positively affect the sensor characteristics of WO_3_ for toluene. Vallejos et al. increased the sensor response (for 20–100 ppm of toluene) of WO_3_ nanoneedles through functionalization with Fe_2_O_3_ and Pt [27], see Figure 3.

By functionalizing WO_3_ nanofibers with Pd and measuring at 350 °C, Kim et al. detected between 1 and 5 ppm in a background of 90% RH [28]. As a result the sensor response to acetone and H_2_S drastically decreased. Choi et al. were able to further increase the positive effect of Pd doping on the sensing characteristics by using a new catalyst functionalization method [29]. Thin-walled Pt and Pd were selectively embedded at the pore sites of electrospun WO_3_. Choi et al. were able to get visible sensor signals to as low as 100 ppb toluene [29]. This was achieved by using polystyrene colloid templates [29].

This concentration range is lower than expected in breath after prolonged exposure to high concentrations of toluene. In fact, it is almost low enough to be suitable for a different sensing objective. Heightened concentrations of toluene are found in patients suffering from lung cancer [28]. The survival rate of lung cancer is very low compared to other cancers because it is often diagnosed in late stages (American Cancer Society Cancer Facts and Figures American Cancer Society). Detection using breath analysis would allow lung cancer to be more easily diagnosed and could potentially raise the survival rate. The exhaled breath of lung cancer patients contains approximately 80–100 ppb of toluene (two- or three-fold higher than the concentrations found in healthy people) [28].

As the understanding of WO_3_-based sensors grows, it may be possible to not only detect exposure to harmful concentrations of toluene but to also screen patients inexpensively for lung cancer. In 2012, Itoh et al. examined how aging affects WO_3_-based sensors’ ability to detect toluene. They found that the signals of non-aged sensors to toluene were humidity-dependent while those that underwent a high-moisture heat treatment (aging) showed stable sensor responses. The moist heat treatment is believed to promote the adsorption of water molecules resulting in the formation of hydroxyl groups that block the adsorption sites of water, therefore, eliminating the effects of humidity [65]. This is an example of how knowledge about how sensing takes place could be useful for optimizing sensors in the future.

The sensing of toluene in human breath via WO_3_-based sensors, both to identify elongated exposure to the toxin and to monitor disease, shows great promise.

### 2.5. Nitrogen Monoxide (NO)

Sampling of breath for NO detection is very difficult because different technical sampling factors can drastically alter the results [66]. Nitrogen monoxide is produced in the body during the enzymatic oxidation of l-arginine [67]. Fractional exhaled NO (FENO) is a biomarker for inflammation [68]. Concentrations of NO over 50 ppb in breath indicate airway eosinophilia [69]. Patients with stable asthma are reported to have NO breath concentrations between 20 and 25 ppb [69]. Normal nasal NO concentrations of children are very high, around 450 ppb [70]. This makes it extremely important to prevent nasal air contamination during sampling [71]. In addition it was found that the ambient NO concentration can be in the ppb range [70]. Due to the high risk of falsified results through contamination, standardization of measurements was critical so that NO measurements could be compared or even reproduced [69]. This standardization is believed to be a key reason why, in 2003, the FDA approved a NO breath detector from Aerocrine (Solna, Sweden) as a medical diagnostic tool [71,72].

The breath sampling used by the Aerocrine detector is highly sophisticated [73]. The inhaled air is scrubbed to prevent contamination through ambient NO. A flow control keeps the exhalation rate constant at 50 mL/s and closure of the velum prevents contamination with nasal NO. The exhalation is set to 10 s and the NO plateau is between 7 s and 10 s [73] to ensure accurate sampling. The NIOX MINO from Aerocrine couples this sampling method with an amperometric liquid electrolyte sensor. Although the device is relatively compact (230 mm × 128 mm × 96 mm) the analyzer costs upwards of $2000 (listing number 1491060 on dotmed.com). For more information please refer to the homepage of Aerocrine. This cost and size could potentially be reduced using a SMOX-based sensor. The use of different WO_3_ based samples appears promising for the detection of breath NO, see Table 8. 

**Figure 4:**
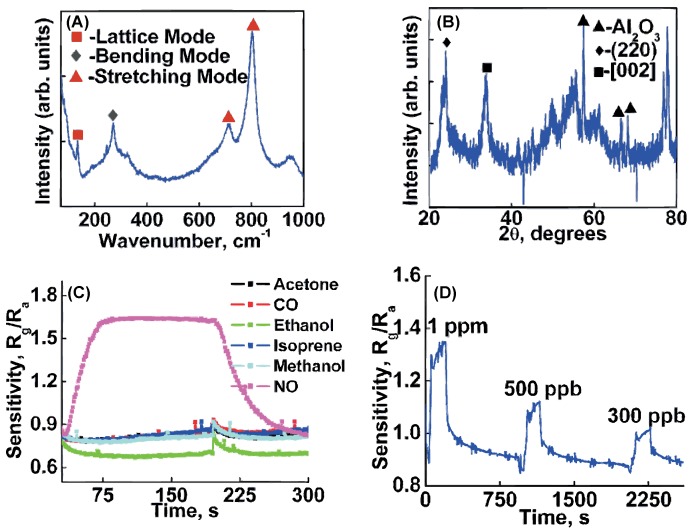
Gouma et al. has found that the gas selectivity is strongly dependent on the crystal phase. (**A**) A raman spectrum taken of the monoclinic γ-WO_3_ sample; (**B**) A XRD spectrum taken of the monoclinic γ-WO_3_ sample; (**C**) Gas sensing response of the monoclinic γ-WO_3_ sample to 10 ppm NO, 10 ppm acetone, 10 ppm isoprene, 50 ppm ethanol, 50 ppm methanol, and 50 ppm CO in synthetic air; (**D**) Gas sensing response of the monoclinic γ-WO_3_ sample to 1 ppm, 500 ppb, and 300 ppb NO in synthetic air. Reprinted from [30] with the permission of AIP Publishing.

Gouma et al. reported that a stoichiometric phase pure (monoclinic) WO_3_ sample showed a high sensor response to NO and low responses to other breath analysis-relevant gases (see Figure 4) [30]. A NO concentration range between 1 ppm and 300 ppb in dry air was examined. Moon et al. reported selective sensors based on 1D villi-like nanostructured WO_3_ with which they could successfully detect down to 200 ppb of NO in a background humidity of 80% RH [31].

In addition to selectivity and the effect of humidity, the effect of NO_2_ on the detection of NO must be considered. During sensing NO is oxidized to NO_2_. Many SMOX-based sensors show a sensor response to NO_2_ [41]. NO is a reducing gas while NO_2_ is an oxidizing gas, meaning the sensor response is opposite. An overlapping sensor response (of equal strength) to NO and NO_2_ would result in no signal or a false negative. Fruhberger et al. addressed this issue by using a permanganate oxidizing agent and a Pt/Al_2_O_3_ catalyst to convert NO to NO_2_ [32]. They then detected the NO_2_ concentration using WO_3_-based sensors. The WO_3_ sample used by Fruhberger et al. showed practically no sensor response to NO (eliminating any cross-sensitivity of the sensor to NO produced during the sensing of NO_2_) [32].

Through standardization it is now possible to sample breath precisely so that even the very low concentrations of NO can be accurately detected. This shows how important it is to understand the origin of the biomarker and sources of possible contamination during sampling. Through proper regulation, breath sampling is a viable option for medical diagnostics. To minimize device cost and size, different possible detectors, for example WO_3_-based sensors will need to be researched.

### 2.6. Hydrogen Sulfide (H_2_S)

Volatile sulfur compounds (VSCs) produced by oral bacteria are responsible for 90% of malodour [74]. Of all of the gases found in human breath VSCs stand out due to their penetrating stench which resembles the smell of rotten eggs. People with bad breath do not notice it themselves, but others are acutely aware of the smell. The concentration of H_2_S in patients suffering from halitosis (bad breath) is ca. 20.6 ppb [36]. Unlike the previously considered biomarkers, the origin of most VSCs in breath is the oral cavity. Recently, breath and in-mouth sampling were compared. Both methods yield similar results for VSCs [75]. The results indicate that sampling using a syringe is adequate for the detection of malodorous compounds in breath [75]. The suitability of WO_3_-based sensors for VSCs is well researched for the example of H_2_S. It has been known for over 20 years that sensors based on WO_3_ show high signals to H_2_S [33]. See Table 9 for an overview of promising WO_3_ samples for the detection of H_2_S. Mixed tetragonal and monoclinic phase samples have been found to be especially suited for the detection of H_2_S. For example, Solis et al. report that the detection of 10 ppm H_2_S at room temperature is possible using sensors based on such mixed-phase samples. The conductance of the film increased three orders of magnitude within 10 min, the very slow recovery time (hours) was increased using a short heating pulse to 530 K for 1 min [76]. Reyes et al. utilized advanced reactive gas deposition to make nanocrystalline films of WO_3_ out of metallic tungsten and oxygen [43]. Overall, Reyes et al. found that ambient oxygen is vital for the detection of H_2_S at low operating temperatures [43]. This information could be useful for tailoring H_2_S sensors in the future. In 2008, Rout et al. even reported that sensors based on WO_2.72_ nanowires are good candidates for sensing concentrations as low as 10 ppm of H_2_S [34]. The response to H_2_S was not significantly affected by humidity under 60% RH [34].

**Figure 5:**
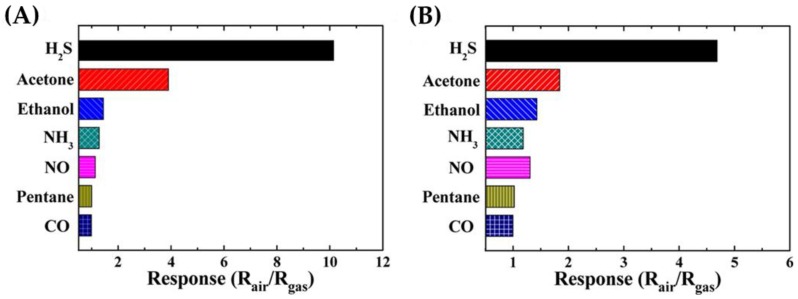
The graphite (0.1 wt%)-WO_3_ (**A**) functionalized hemitubes showed high sensor signals to 2 ppm H_2_S than the graphene oxide (0.1 wt%)-WO_3_ (**B**) functionalized hemitubes. All test gases were measured at 2 ppm and a background humidity of 85%–95%. The figure is reprinted with permission from [35].

Using sensors based on graphite (GR)- and graphene oxide (GO)-functionalized WO_3_ hemitubes, Choi et al. reported increased sensor signals and were also able to detect levels as low as 1 ppm of H_2_S [35] (see Figure 5). The sensors based on graphite (0.1 wt%)-WO_3_ functionalized hemitubes showed higher signals than those containing graphene oxide (see Figure 5). The sensor signals to acetone, however, also increased as a result of functionalization, indicating limited selectivity [35]. Sekhar et al. found that by using WO_3_ nanoplatelets, they could attain a high sensor response to just 1 ppm of H_2_S. At higher concentrations they reported that variations in background humidity had a negligible effect on the sensor response [34].

In addition to H_2_S, other VSCs, such as methyl mercaptan and dimethyl sulfide, are responsible for bad breath [77]. This makes breath analysis difficult for halitosis due to cross-sensitivity. Today commercial options exist, for example the OralChroma™ device from Fis Inc. (Hyogo, Japan) that couples a small gas chromatograph with SMOX-based sensors. Choi et al. used Pt-infiltrated block copolymer microparticles to functionalize macroporous WO_3_ nanofibers. They created a sensor array containing sensors based on pristine WO_3_, 0.042 wt% Pt-WO_3_, and 0.008 wt% Pt-WO_3_. Using principal component analysis, Choi et al. were able to distinguish H_2_S, methyl mercaptan, acetone, and toluene within a breath analysis relevant concentration range (1–5 ppm) [78]. Using a sensor array it may be possible to accurately detect halitosis in the future. The next vital step will be to examine what effect the different VSCs have on SMOX-based sensors. Only then can sensors be selected. In the case of an array in addition to examining each single sensor the ideal interplay between sensors must also be achieved.

In the future, perhaps through morphology control, doping, and a clever combination of WO_3_-based sensors, chromatographs may no longer be necessary to detect halitosis.

## 3. Conclusions

It has been known since antiquity that human breath contains information about a person’s wellbeing. As analytical methods developed, it became possible to identify, and even quantify, gaseous components in breath. As the detection methods became more precise, it was recognized that, for breath analysis, more exact sampling methods were necessary. In the last few years great progress has been made in breath sampling and, now accurate modular samplers exist for different biomarkers. Today accurate sampling and detection is possible. The next step in breath analysis will be towards miniaturization and cost reduction. For this, SMOX-based sensors are an attractive option because they are small, inexpensive, robust, and sensitive.

In particular, WO_3_-based sensors show promise for implementation in breath analysis. WO_3_ is already a widely used material for commercially available SMOX-based sensors. A survey of existing research revealed that WO_3_ shows high sensor responses to several breath analysis-relevant gases, e.g., acetone, H_2_S, and toluene. Modern material science methods for the synthesis of WO_3_ show promise for increasing the selectivity and sensitivity of sensors. Through functionalization with different additives it was possible to tailor different WO_3_ materials for the detection of certain gases within specific concentration ranges. Despite promising results within laboratory settings for different WO_3_ samples, several issues continue to remain unresolved, in particular, cross-sensitivity to water and other gases in breath. Using existing modular breath samplers WO_3_-based sensors can be tested in application conditions, allowing the accuracy and precision of WO_3_-based sensors to be compared to more expensive analytical methods. Through the continued advances of material science more modification possibilities will be developed to tailor WO_3_ samples. By better understanding how sensing works and what factors play a role, WO_3_ materials can be strategically altered. For example certain crystal phases appear to be better suited for certain gases. This basic understanding of sensing with WO_3_ is still relatively limited and will need to be further expanded in the future. Once the sensors are comparable in quality to more sophisticated analytical methods, they can be used for breath analysis.

In summary, as the digitalization era continues, and the application of sensors becomes more widespread, the role of SMOX sensors will increase. In this review, the viability of WO_3_-based sensors for breath analysis was explored. Although currently no WO_3_-based sensors are commercially available for medical diagnostics, their use in the future seems highly promising. The use of WO_3_ based sensors will allow for the production of small, inexpensive, and portable health screening devices.

## Figures and Tables

**Table 1:** In this table different breath analysis relevant gases which have reportedly been detected using WO_3_-based sensors are listed.

Gas	Biomarker Origin	Concentration
Ethanol [9,10,11]	Blood Alcohol Concentration	>130–650 ppm [12]
Acetone [13,14,15,16,17,18]	Diabetes	up to 3.73 ppm [19]
NH_3_ [20,21,22,23,24]	Renal Failure	around 4.8 ppm [25]
Toluene [26,27,28,29]	Exposure to Toxin	>56 ppm
	Lung Cancer	80–100 ppb [28]
Nitric oxide [30,31,32]	Asthma	>30 ppb [31]
H_2_S [33,34,35]	Halitosis	>20.6 ppb [36]

**Table 2:** WO_3_ has several different stable crystal phases [38,48].

Crystal Phase	Symmetry	Temperature
ε*-*WO_3_	Monoclinic	Under −40 °C
δ-WO_3_	Triclinic	−40–17 °C
γ-WO_3_	Monoclinic	17–320 °C
β-WO_3_	Orthorhombic	320–720 °C
α-WO_3_	Tetragonal	Over 720 °C
h-WO_3_	Hexagonal	Metastabile

**Table 3:** The table shows a comparison of different accuracies/precisions to price/detector type. The higher the accuracy value and the lower the precision value, the better the detector.

Manufacturer	Model	Detector Type	Price in €	Accuracy [52]	Precision [52]
ACE	Neo	Fuel Cell	~99 ^a^	95.7%	2.6%
ACE	II Basic	Fuel Cell	~110 ^a^	99.0%	1.3%
ACE	III Basic	Fuel Cell	~150 ^a^	95.6%	2.6%
ACE	III Premium	Fuel Cell	~160 ^a^	86.2%	29.7%
ACE	AF-33	Fuel Cell	~100 ^a^	97.9%	2.6%
ACE	Alco 5500	SMOX	~36 ^a^	96.2%	17.5%
ACE	Pro Med Basic	Fuel Cell	~350 ^b^	88.5%	20.6%
ACE	One	Fuel Cell	~120 ^a^	98.6%	2.0 %
Draeger	Alcotest 3000	Fuel Cell	~300 ^a^	98.9 %	2.7%
ACE	Public V	Fuel Cell	~1400 ^a^	98.0%	2.8%
ACE	Stationary Alcohol Analyzer	Fuel Cell	Not Found	77.1%	1.4%

^a^ Price is that listed on www.Amazon.de on 21 September 2016; ^b^ Price found on http://www.alkomat-shop.at on 21 September 2016.

**Table 4:** A list of different samples used to detect ethanol.

Sample	Nanostructure	Crystal Structure	Ethanol Concentration
Au:WO_3_ [9]	Nanoparticles 40 nm (Crystallites)	Unknown	2% in Dry Air
WO_3_ [10]	Nanorods 50 nm (Diameter)	Monoclinic WO_3_	12.5–31.25 ppm
WO_3_-SnO_2_ [11]	Nanoplates 40–400 nm (Length) 20–60 nm (Thickness)	Monoclinic WO_3_	180–2800 ppm
Au-NP Decorated WO_3_ [56]	Nanowires ~5 μm (Length) 60–120 nm (Diameter)	Unknown	With UV Irradiation 25–75 ppm

**Table 5:** A list of different samples used to detect acetone.

Sensor	Nanostructure	Crystal Structure	Acetone Concentration
10 mol% Si:WO_3_ [16]	12 nm	Monoclinic ε-WO_3_	100–600 ppb in Dry Air to 90% RH
WO_3_ [14]	Hollow Spheres 400 nm (Diameter) 30 nm (Shell) 12 nm (Crystallites)	Monoclinic (JCPDS card no. 72-0677	50–500 ppm
Rh-Loaded WO_3_ [15]	Hollow Spheres 25 nm (Shell)	Orthorhombic β-WO_3_	0.2–20 ppm in Dry Air up to 80% RH
20 at% Cr-Doped WO_3_ [17]	Nanoparticles 20 nm (Crystallites)	ε-WO3	0.2–1 ppm
0.022 wt% Pt Loaded WO_3_ [18]	Nanoparticles 28 nm (Crystallites)	Not Defined	0.1–5 ppm

**Table 6:** A list of different samples used to detect ammonia.

Sensor	Nanostructure	Crystal Structure	Ammonia Concentration
0.4 wt% Au loaded WO_3_ [20]	Unknown	Unknown	0.001–100 ppm
WO_3_ [21]	Undefined	Unknown	10–1000 ppm in dry air
WO_3_ [22]	Carnation 500 nm (Length) 80 nm (Diameter)	Hexagonal h-WO_3_	10–100 ppm
WO_3_ [23]	Nanorod 30–100 nm (Diameter) 100–300 nm (Length) Nanoparticles 10–40 nm	Hexagonal h-WO_3_	80–200 ppm
Cu and V Modified WO_3_ [24]	Nanoparticles 19–64 nm	Monoclinic γ-WO_3_ and Triclinic δ-WO_3_	500 ppm

**Table 7:** A list of different samples used to detect toluene.

Sensor	Nanostructure	Crystal Structure	Toluene Concentration
WO_3_·H_2_O [26]	Nanoplates 20–30 nm (Thickness) 150–200 nm (Length)	Orthorhombic β-WO_3_	10–200 ppm
Fe_2_O_3_@WO_3__‑__x_ [27]	Nanoneedles 50–100 nm (Diameter) ~10µm (Length)	Monoclinic ((P21/n), ICCD card no. 72-0677)	20–100 ppm
0.5 wt% Pd-nanoparticles/0.5 wt% Pd-embedded WO_3_ [28]	Nanofibers 300–600 nm (Diameter) 17.4–32.59 nm (Crystallites)	Monoclinic (PDF#43-1035)	1–5 ppm
Pd loaded WO_3_ [29]	Nanofibers 200–300 nm (Diameter)	Unknown	0.1–5 ppm in 90% RH

**Table 8:** A list of different samples used to detect NO.

Sensor	Nanostructure	Crystal Structure	NO Concentration
WO_3_ [30]	Nanoparticles 15–20 nm	Monoclinic γ-WO_3_	0.3–1 ppm
WO_3_ [31]	Villi (Single Crystalline) 40–50 nm (Diameter)	Monoclinic WO_3_	0.2–1 ppm in 80% RH
WO_3_ with a Filter and Oxidizing Agent (KMnO_4_) [32]	Unknown	Unknown	60 ppb

**Table 9:** A list of different samples used to detect H_2_S.

Sensor	Grain Size	Crystal Structure	H_2_S Concentration
WO_3_ [33]	Unknown	Unknown	10–100 ppm
WO_2.72_ [34]	Nanowires (Single Crystal) 5–15 nm	Monoclinic (JCPDS no: 36-101)	1–1000 ppm
Graphene Functionalized WO_3_ [35]	Hemitubes 200–300 nm (Diameters)	Monoclinic (JCPDS no: 43-1035)	1–5 ppm
WO_3_ [76]	Nanoparticles ~11 nm (Crystallites)	Monoclinic and Tetragonal WO_3_	10 ppm
WO_3_ [43]	Nanoparticles ~10 nm (Crystallites)	Monoclinic and Tetragonal	10 ppm
Pt Functionalized WO_3_ [78]	Nanofiber ~900 nm (Diameter)	Unknown	1–5 in Dry Air to 95% RH

## References

[1] Dweik R., Amann A. (2008). NIH Public Access. J Breath Res..

[2] Pauling L., Robinson A.B., Teranishi R., Cary P. (1971). Quantitative analysis of urine vapor and breath by gas-liquid partition chromatography. Proc. Natl. Acad. Sci. USA.

[3] Risby T.H., Pleil J.D. (2013). Breath analysis-past, present and future: A special issue in honour of Michael Phillips’ 70th birthday. J. Breath Res..

[4] Phillips M., Greenberg J., Sabas M. (1994). Alveolar gradient of pentane in normal human breath. Free Radic. Res..

[5] Drobek M., Kim J.H., Bechelany M., Vallicari C., Julbe A., Kim S.S. (2016). MOF-Based Membrane Encapsulated ZnO Nanowires for Enhanced Gas Sensor Selectivity. ACS Appl. Mater. Interfaces.

[6] Liu Y., Yao S., Yang Q., Sun P., Gao Y., Liang X., Liu F., Lu G. (2015). Highly sensitive and humidity-independent ethanol sensors based on In_2_O_3_ flower/SnO_2_ nanoparticle composites. RSC Adv..

[7] Righettoni M., Amann A., Pratsinis S.E. (2015). Breath analysis by nanostructured metal oxides as chemo-resistive gas sensors. Mater. Today.

[8] Righettoni M., Tricoli A., Gass S., Schmid A., Amann A., Pratsinis S.E. (2012). Breath acetone monitoring by portable Si:WO_3_ gas sensors. Anal. Chim. Acta.

[9] Labidi A., Gillet E., Delamare R., Maaref M., Aguir K. (2006). Ethanol and ozone sensing characteristics of WO_3_ based sensors activated by Au and Pd. Sens. Actuators B Chem..

[10] Ahmad M.Z., Kang J.H., Sadek A.Z., Moafi A., Sberveglieri G., Wlodarski W. (2012). Synthesis of WO_3_ nanorod based thin films for ethanol and H_2_ Sensing. Procedia Eng..

[11] Nayak A.K., Ghosh R., Santra S., Guha P.K., Pradhan D. (2015). Hierarchical Nanostructured WO_3_-SnO_2_ for Selective Sensing of Volatile Organic Compounds. Nanoscale.

[12] Mitsubayashi K., Yokoyama K., Takeuchi T., Karube I., Takeuchl T. (1994). Gas-Phase Biosensor for Ethanol. Anal. Chem..

[13] Righettoni M., Ragnoni A., Güntner A.T., Loccioni C., Pratsinis S.E., Risby T.H. (2015). Monitoring breath markers under controlled conditions. J. Breath Res..

[14] Li X.-L., Lou T.-J., Sun X.-M., Li Y.-D. (2004). Highly Sensitive WO_3_ Hollow-Sphere Gas Sensors. Inorg. Chem..

[15] Choi K.-I., Hwang S.-J., Dai Z., Kang Y.C., Lee J.-H. (2014). Rh-catalyzed WO_3_ with anomalous humidity dependence of gas sensing characteristics. RSC Adv..

[16] Righettoni M., Tricoli A., Pratsinis S.E. (2010). Si:WO_3_ Sensors for Highly Selective Detection of Acetone for Easy Diagnosis of Diabetes by Breath Analysis. Anal. Chem..

[17] Wang L., Teleki A. (2008). Ferroelectric WO_3_ Nanoparticles for Acetone Selective Detection. Chem. Mater..

[18] Kim S.-J., Choi S.-J., Jang J.-S., Kim N.-H., Hakim M., Tuller H.L., Kim I.-D. (2016). Mesoporous WO_3_ Nanofibers with Protein Templated Nanoscale Catalysts for Detection of Trace Biomarkers in Exhaled Breath. ACS Nano.

[19] Deng C., Zhang J., Yu X., Zhang W., Zhang X. (2004). Determination of acetone in human breath by gas chromatography-mass spectrometry and solid-phase microextraction with on-fiber derivatization. J. Chromatogr. B Anal. Technol. Biomed. Life Sci..

[20] Maekawa T., Tamaki J., Miura N., Yamazoe N. (1992). Gold-Loaded Tungsten Oxide Sensor for Detection of Ammonia in Air. Chem. Lett..

[21] Llobet E., Molas G., Molinàs P., Calderer J., Vilanova X., Brezmes J., Sueiras J.E., Correig X. (2000). Fabrication of Highly Selective Tungsten Oxide Ammonia Sensors. J. Electrochem. Soc..

[22] Takács M., Dücső C., Lábadi Z., Pap A.E. (2014). Effect of hexagonal WO_3_ morphology on NH_3_ sensing. Proc. Eng..

[23] Wang L., Pfeifer J., Balázsi C., Gouma P.I. (2007). Synthesis and Sensing Properties to NH_3_ of Hexagonal WO_3_ Metastable Nanopowders. Mater. Manuf. Process..

[24] Jiménez I., Centeno M.A., Scotti R., Morazzoni F., Cornet A., Morante J.R. (2003). NH_3_ Interaction with Catalytically Modified Nano-WO_3_ Powders for Gas Sensing Applications. J. Electrochem. Soc..

[25] Davies S., Spanel P., Smith D. (1997). Quantitative analysis of ammonia on the breath of patients in end-stage renal failure. Kidney Int..

[26] Miao B., Zeng W., Mu Y., Yu W., Hussain S., Xu S., Zhang H., Li T. (2015). Controlled synthesis of monodisperse WO_3_·H_2_O square nanoplates and their gas sensing properties. Appl. Surf. Sci..

[27] Vallejos S., Gracia I., Figueras E., Cane C. (2015). Nanoscale Heterostructures Based on Fe_2_O_3_@WO_3−x_ Nanoneedles and Their Direct Integration into Flexible Transducing Platforms for Toluene Sensing. ACS Appl. Mater. Interfaces.

[28] Kim N.H., Choi S.J., Yang D.J., Bae J., Park J., Kim I.D. (2014). Highly sensitive and selective hydrogen sulfide and toluene sensors using Pd functionalized WO_3_ nanofibers for potential diagnosis of halitosis and lung cancer. Sens. Actuators B Chem..

[29] Choi S.-J., Kim S.-J., Koo W.-T., Cho H.-J., Kim I.-D. (2015). Catalyst-loaded porous WO_3_ nanofibers using catalyst-decorated polystyrene colloid templates for detection of biomarker molecules. Chem. Commun. (Camb.).

[30] Gouma P.I., Kalyanasundaram K. (2008). A selective nanosensing probe for nitric oxide. Appl. Phys. Lett..

[31] Moon H.G., Choi Y.R., Shim Y.S., Choi K.I., Lee J.H., Kim J.S., Yoon S.J., Park H.H., Kang C.Y., Jang H.W. (2013). Extremely sensitive and selective NO probe based on villi-like WO_3_ nanostructures for application to exhaled breath analyzers. ACS Appl. Mater. Interfaces.

[32] Fruhberger B., Stirling N., Grillo F.G., Ma S., Ruthven D., Lad R.J., Frederick B.G. (2001). Detection and quantification of nitric oxide in human breath using a semiconducting oxide based chemiresistive microsensor. Sens. Actuators B Chem..

[33] Xu Z., Vetelino J.F., Lec R., Parker D.C. (1990). Electrical properties of tungsten trioxide films. J. Vac. Sci. Technol. A Vac. Surf..

[34] Rout C.S., Hegde M., Rao C.N.R. (2008). H_2_S sensors based on tungsten oxide nanostructures. Sens. Actuators B Chem..

[35] Choi S.J., Fuchs F., Demadrille R., Grevin B., Jang B.H., Lee S.J., Lee J.H., Tuller H.L., Kim I.D. (2014). Fast responding exhaled-breath sensors using WO_3_ hemitubes functionalized by graphene-based electronic sensitizers for diagnosis of diseases. ACS Appl. Mater. Interfaces.

[36] Tangerman A., Winkel E.G. (2007). Intra- and extra-oral halitosis: Finding of a new form of extra-oral blood-borne halitosis caused by dimethyl sulphide. J. Clin. Periodontol..

[37] Christian J., Gaur R.P.S., Wolfe T., Trasorras J.R.L. (2011). Tungsten Chemicals and Their Applications.

[38] Zheng H., Ou J.Z., Strano M.S., Kaner R.B., Mitchell A., Kalantar-Zadeh K. (2011). Nanostructured tungsten oxide-properties, synthesis, and applications. Adv. Funct. Mater..

[39] Long H., Zeng W., Zhang H. (2015). Synthesis of WO_3_ and its gas sensing: A review. J. Mater. Sci. Mater. Electron..

[40] Hübner M., Simion C.E., Haensch A., Barsan N., Weimar U. (2010). CO sensing mechanism with WO_3_ based gas sensors. Sens. Actuators B Chem..

[41] Akamatsu T., Itoh T., Izu N., Shin W. (2013). NO and NO_2_ sensing properties of WO_3_ and Co_3_O_4_ based gas sensors. Sensors.

[42] Baumann S.L. (2003). Detektions-Mechanismen Auf WO_3_ Bei Einsatz in Verbrennungsabgasen.

[43] Reyes L.F., Saukko S., Hoel A., Lantto V., Granqvist C.G. (2004). Structure engineering of WO_3_ nanoparticles for porous film applications by advanced reactive gas deposition. J. Eur. Ceram. Soc..

[44] Barsan N., Weimar U. (2001). Conduction model of metal oxide gas sensors. J. Electroceramics.

[45] Weil M., Schubert W. (2013). The Beautiful Colours of Tungsten Oxides.

[46] Pokhrel S., Birkenstock J., Dianat A., Zimmermann J., Schowalter M., Rosenauer A., Ciacchi L.C., Mädler L. (2015). In situ high temperature X-ray diffraction, transmission electron microscopy and theoretical modeling for the formation of WO_3_ crystallites. CrystEngComm.

[47] Xin G., Guo W., Ma T. (2009). Effect of annealing temperature on the photocatalytic activity of WO_3_ for O_2_ evolution. Appl. Surf. Sci..

[48] Gerand B., Nowogrocki G., Guenot J., Figlarz M. (1979). Structural study of a new hexagonal form of tungsten trioxide. J. Solid State Chem..

[49] Faller A., Michael S. (2016). Der Körper des Menschen: Einführung in Bau und Funktion.

[50] Measuring Alcohol in the Body.

[51] Lemaire N. (2005). Technical Evaluation: ACS and Dräger Breath Alcohol Ignition Interlock Devices (BAIIDs).

[52] Rosenberg E. (2015). A Study of the Accuracy and Precision of Selected Breath Alcohol Measurement Devices (‘Breathalyzers’).

[53] Santra S., Sinha A.K., de Luca A., Ali S.Z., Udrea F., Guha P.K., Ray S.K., Gardner J.W. (2016). Mask-less deposition of Au–SnO_2_ nanocomposites on CMOS MEMS platform for ethanol detection. Nanotechnology.

[54] Kumar A. (2015). Performance analysis of zinc oxide based alcohol sensors. J. Appl. Sci. Eng. Res..

[55] Comini E. (2016). Metal oxide nanowire chemical sensors: Innovation and quality of life. Mater. Today.

[56] Trawka M., Smulko J., Hasse L., Granqvist C.G., Annanouch F.E., Ionescu R. (2016). Fluctuation enhanced gas sensing with WO_3_-based nanoparticle gas sensors modulated by UV light at selected wavelengths. Sens. Actuators B Chem..

[57] Neri G. (2015). First Fifty Years of Chemoresistive Gas Sensors. Chemosensors.

[58] Rooth G., Ostenson S. (1966). Acetone in alveolar air, and the control of diabetes. Lancet.

[59] Essiet I. (2013). Diagnosis of kidney failure by analysis of the concentration of ammonia in exhaled human breath. J. Emerg. Trends Eng. Appl. Sci..

[60] Epifani M., Andreu T., Magana C.R., Diaz R., Arbiol J., Siciliano P., Morante J.R. (2010). From doping to phase transformation: Ammonia sensing performances of chloroalkoxide-derived WO_3_ powders modified with chromium. Sens. Actuators B Chem..

[61] De Boer M., van Dillen A.J., Koningsberger D.C., Janssen F.J.J.G., Koerts T., Geus J.W. (1992). Selective oxidation of ammonia to nitrogen over silica supported molybdena catalysts. A structure-selectivity relationship. Stud. Surface Sci. Catal..

[62] Solga S.F., Mudalel M., Spacek L.A., Lewicki R., Tittel F., Loccioni C., Russo A., Risby T.H. (2013). Factors influencing breath ammonia determination. J. Breath Res..

[63] Li F., Li C., Zhu L., Guo W., Shen L., Wen S., Ruan S. (2016). Enhanced toluene sensing performance of gold-functionalized WO_3_·H_2_O nanosheets. Sens. Actuators B Chem..

[64] Carlsson A. (1982). Exposure to toluene. Uptake, distribution and elimination in man. Scand. J. Work. Environ. Heal..

[65] Itoh T., Matsubara I., Tamaki J., Kanematsu K. (2012). Effect of High-Humidity Aging on Performance of Tungsten Oxide-Type Aromatic Compound Sensors. Sens. Mater..

[66] Barnes P.J., Kharitonov S.A. (1996). Exhaled nitric oxide: A new lung function test. Thorax.

[67] Morris S.M., Billiar T.R. (1994). New insights into the regulation of inducible nitric oxide synthesis. Am. J. Physiol..

[68] Gouma P., Sood S., Stanacevic M., Simon S. (2014). Selective chemosensing and diagnostic breathanalyzer. Procedia Eng..

[69] Dweik R.A., Boggs P.B., Erzurum S.C., Irvin C.G., Leigh M.W., Lundberg J.O., Olin A., Plummer A.L., Taylor D.R., Thoracic A. (2011). An Official ATS Clinical Practice Guideline: Interpretation of Exhaled Nitric Oxide Levels (FENO) for Clinical Applications. Am. J. Respir. Crit. Care Med..

[70] Struben V.M.D., Wieringa M.H., Mantingh C.J., Bommeljé C., Don M., Feenstra L., de Jongste J.C. (2005). Nasal NO: Normal values in children age 6 through to 17 years. Eur. Respir. J..

[71] Offline N.O. (1999). Recommendations for Standardized Procedures for the Online and Offline Measurement of Exhaled Lower Respiratory Nitric Oxide and Nasal Nitric Oxide in Adults and Children—1999. Am. J. Respir. Crit. Care Med..

[72] Paschke K.M., Mashir A., Dweik R.A. (2010). Clinical applications of breath testing. Med. Rep..

[73] Aerocrine (2007). Mastering the Measurement of Feno Nitric Oxide Monitoring System NIOX^®^ Flex—Nitric Oxide Monitoring System.

[74] Washio J., Sato T., Koseki T., Takahashi N. (2005). Hydrogen sulfide-producing bacteria in tongue biofilm and their relationship with oral malodour. J. Med. Microbiol..

[75] Paetznick D.J., Reineccius G.A., Peppard T.L., Herkert J.M., Lenton P. (2010). Comparison of breath and in-mouth collection for the measurement of oral malodorous compounds by gas chromatography using sulfur chemiluminescence detection. J. Breath Res..

[76] Solis J.L., Hoel A., Kish L.B., Granqvist C.G. (2001). Gas-Sensing Properties of Nanocrystalline WO_3_ Films Made by Advanced Reactive Gas Deposition. J. Am. Ceram. Soc..

[77] De Geest S., Laleman I., Teughels W., Dekeyser C., Quirynen M. (2016). Periodontal diseases as a source of halitosis: A review of the evidence and treatment approaches for dentists and dental hygienists. Periodontol. 2000.

[78] Choi S.-J., Ku K.H., Kim B.J., Kim I.-D. (2016). Novel templating route using Pt infiltrated block copolymer micro-particles for catalytic Pt functionalized macroporous WO_3_ nanofibers and its application in breath pattern recognition. ACS Sens..

